# Two Cases of Highly Symptomatic Interarterial Anomalous Right Coronary Arteries

**DOI:** 10.7759/cureus.42761

**Published:** 2023-07-31

**Authors:** Kyle J Clay, Arsalan Hamid, Bradley P Deere

**Affiliations:** 1 Department of Medicine, University of Mississippi Medical Center, Jackson, USA; 2 Department of Cardiovascular Diseases, University of Mississippi Medical Center, Jackson, USA

**Keywords:** coronary unroofing, adult congenital heart disease, anomalous coronary artery origin, cardiac stress test, coronary artery angiography, cardiac chest pain, anomalous origin of right coronary artery, right coronary artery (rca), coronary computed tomoangiography, coronary vessel anomaly

## Abstract

Coronary artery anomalies are a broad group of congenital coronary artery variations. Anomalous aortic origin of a coronary artery is a variant that occurs when a coronary artery arises from an inappropriate sinus of Valsalva. While most patients are asymptomatic, these congenital variants may predispose them to symptoms or even sudden cardiac death (SCD). Unfortunately, no unified consensus exists on risk stratification or management of patients with these congenital variants. We present two unique cases of symptomatic anomalous right coronary arteries and discuss their presentations, imaging findings, and management.

## Introduction

Coronary artery anomalies are a broad group of congenital structural variations involving the three main coronary arteries. These anomalies are one of the most frequently encountered congenital cardiac variations [1]. The reported population statistics of coronary artery anomalies vary, with one of the largest angiographic studies suggesting the incidence is around 1.3% [2]. A recent review suggests that interarterial anomalous right coronary arteries are less frequently encountered, with a weighed prevalence of 0.23% [3]. While most of these variants are benign, they also represent the second most common cause of sudden cardiac death (SCD) in young athletes, after hypertrophic cardiomyopathy [4]. While SCD is more frequently encountered in anomalous left coronary arteries from the right sinus of Valsalva, there are reported cases of SCD in patients with anomalous right coronary arteries from the left sinus of Valsalva [5,6]. The traditional pathophysiologic mechanism of ischemia was hypothesized to be due to compression between the great vessels during exercise. However, the actual mechanism is likely more complex. In addition, specific structural and morphological features of these variants can increase the risk of SCD and present challenges for clinicians. This article discusses two unique presentations of anomalous right coronary arteries, imaging findings, work-up, and management.

## Case presentation

Case 1

**Figure 1:**
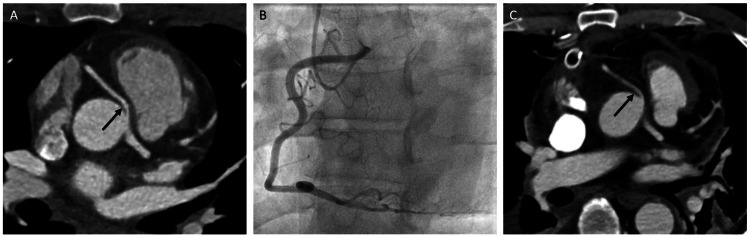
Imaging findings Axial contrasted CT scan (A) of the chest showing an anomalous RCA arising from the left sinus of Valsalva coursing between the aorta and right ventricular outflow tract. Coronary angiography (B) showing the RCA with an anomalous takeoff from the left coronary cusp. Post-operative axial contrasted CT scan (C) of the chest showing a widely patent RCA

A 54-year-old male with hypertension and hyperlipidemia presented to the emergency department with severe substernal chest pain and palpitations. He was hypertensive and had no significant findings on his physical exam. EKG showed normal sinus rhythm without ST-segment or T-wave abnormalities. His serial high-sensitivity troponins were negative. A CT angiogram of the chest was negative for aortic dissection. However, it showed an anomalous right coronary artery (RCA) arising from the left sinus of Valsalva coursing between the aorta and right ventricular outflow tract with 50% luminal narrowing of the first 10 mm suggesting an intramural course (Figure 1a). He was referred to outpatient cardiology for further work-up. In the early stages of his exercise stress EKG, he experienced dyspnea and dizziness. Coronary angiography confirmed an RCA with anomalous takeoff from the left coronary cusp and no significant coronary artery disease (Figure 1b). Because of the severity of his symptoms, he was referred to adult congenital heart surgery and underwent coronary artery unroofing. The RCA was confirmed to have an acute takeoff angle with an intramural course and slit-like origin during the procedure. He tolerated the procedure well without complication. Post-operative imaging showed a widely patent RCA (Figure 1c). His chest pain improved significantly after the procedure.

Case 2

**Figure 2:**
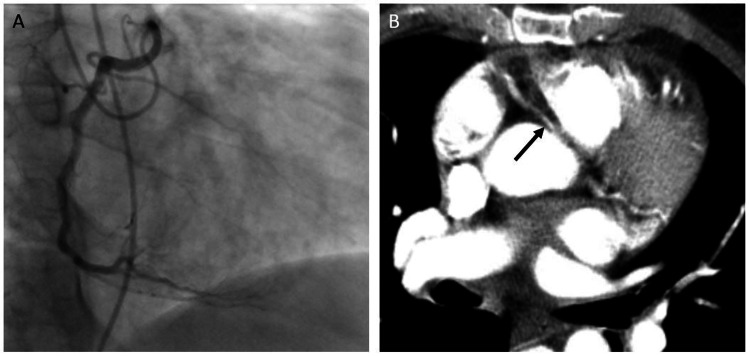
Imaging findings Coronary angiography (A) showing an anomalous RCA off the left coronary cusp with high anterior takeoff. Axial contrasted CT scan (B) of the chest showing an anomalous RCA arising from the left sinus of Valsalva coursing between the aorta and pulmonary artery with the proximal RCA with an oval/slit-like appearance suggesting an intramural course

A 53-year-old male with hypertension, type 1 diabetes, peripheral arterial disease, and end-stage renal disease on hemodialysis arrived at the emergency department with chest pain. The pain woke him up, and it was severe, substernal, and constant. Physical exam was notable for trace ankle edema. EKG showed normal sinus rhythm, an incomplete right bundle branch block, and no ST-segment or T-wave abnormalities. His troponin was elevated to 0.37 (normal 0.00-0.04 ng/mL). Due to concern for a non-ST-elevation myocardial infarction, he was taken for coronary angiography. Coronary angiography showed no significant coronary artery disease but did show an anomalous RCA from the left coronary cusp with high anterior takeoff, compromising the ostial lumen (Figure 2a). In addition, he was noted to have severe compression of the proximal vessel during ventricular systole, supporting a likely course of the vessel between the aorta and pulmonary artery. CT of the chest was performed and noted the anomalous RCA with an intramuscular course (Figure 2b). It was felt that his chest pain was likely secondary to his anomalous RCA. Cardiothoracic surgery evaluated the patient; however, he was unable to proceed with a procedure at that time. He was treated medically with beta-blocker therapy (Lopressor 25 mg BID) and close monitoring. On follow-up, his chest pain was relatively infrequent.

## Discussion

Coronary artery anomalies are diverse groups of congenital coronary artery variations. They are broadly categorized by anomalies of origin (e.g., opposite coronary sinus, pulmonary artery), anomalies of course (e.g., interarterial, myocardial bridging), or anomalies of termination (e.g., fistula).

Anomalous aortic origin of a coronary artery (AAOCA) is a coronary artery that arises from an abnormal sinus of Valsalva or another vessel (e.g., a pulmonary artery). Numerous anatomic variations exist and are often grouped based on their proclivity to cause symptoms or an increased risk of SCD. Early observational studies characterized potentially "lower risk" variants by comparing angiographic studies and patient symptoms [2]. Some of these variants included a left anterior descending or a left circumflex (LCx) arising from separate ostia with an absent left main (LM), LCx arising from the RCA or right sinus of Valsalva, ectopic LM or RCA from the posterior sinus of Valsalva, or small coronary fistulas [2]. These anomalies can usually be monitored and do not require intervention. On the other end of the spectrum are the "higher risk" variants, such as an anomalous left or RCA arising from the pulmonary artery. Surgical intervention is almost always indicated with these variants [1]. The middle of this spectrum lies the anomalous aortic origin of a coronary artery from the opposite sinus of Valsalva, specifically the left coronary artery arising from the right coronary sinus (L-AAOCA) or RCA arising from the left coronary sinus (R-AAOCA). These prove more challenging to risk stratify and envelop most research and literature. These anomalies can take various anatomic courses from their origin, including interarterial (between the aorta and pulmonary artery), subpulmonic, pre-pulmonic, retroaortic, or retrocardiac [3].

Most AAOCA are discovered incidentally upon evaluation for other etiologies that cause cardiac symptoms. While these variants can cause SCD at younger ages, more often, they cause exertional chest pain, dyspnea, arrhythmia, or syncope. However, most patients are completely asymptomatic. The differential diagnosis when evaluating patients with these complaints is vast, including coronary artery disease, pulmonary embolism, and aortic dissection, or in younger patients may include hypertrophic obstructive cardiomyopathy. While these variants are rare, with increased use of imaging, they are being detected more frequently. Various incidence ranges are reported in the literature from autopsy and observational studies. One of the most extensive studies examined over 100,000 patients undergoing coronary angiography and noted an incidence of 1.3% [2]. Recently, Cheezum et al. completed a broad review that showed a weighted prevalence of interarterial R-AAOCA of 0.23% of AAOCA, notably more common than interarterial L-AAOCA (0.03% weighed prevalence) [3].

When risk stratifying patients with AAOCA, assessing high-risk anatomical features is essential. These include an interarterial course, intramural course, slit-like ostium, proximal narrowing of the vessel, and an acute takeoff angle [7]. These features could be related to ischemia leading to worsening symptoms or increased risk of SCD [6]. The pathophysiological mechanism of ischemia/SCD in AAOCA is highly debated in the medical literature, with recent advances giving additional insights. Previously, the universally accepted mechanism of ischemia was related to compression between the pulmonary trunk and aorta during exercise. However, there is likely more at play, given the lower pulmonary artery pressure. Particularly the high-risk anatomical features described previously seem to play a significant role. In addition, there is likely interplay between fixed and dynamic components of AAOCA [8]. According to Bigler et al., the fixed component includes the slit-like ostium and proximal narrowing of the vessel that restricts coronary flow [8]. The dynamic component is primarily affected by the intramural segment and compression from the aorta during exercise, particularly with changes in stroke volume, heart rate, and blood pressure [8].

Multiple imaging modalities can be used to access anomalous coronary arteries. Class 1 recommendations include coronary angiography, CT, or cardiac magnetic resonance (CMR) to evaluate anomalous coronary arteries [1]. Coronary computed tomography angiography (CCTA) has become the gold imaging standard. It has excellent spatial resolution and can produce 3D reformatted images delineating high-risk features, notably an intramural course [9]. CMR can be used, albeit with slightly lower spacial resolution than CCTA [3]. However, late gadolinium enhancement can identify areas of scar that may be arrhythmogenic substrates in the event of repeated myocardial ischemia during exercise [6,10]. These areas of scar may be used for further risk stratification. Echocardiogram can also be used, although it is technician dependent. Finally, coronary angiography can be helpful to assess these anomalies. While it still holds a Class 1 indication, CCTA offers better spatial resolution [1]. However, with the addition of fractional flow reserve (FFR) and intravascular ultrasound (IVUS), angiography can allow for better functional assessment [11].

Accessing myocardial ischemia is another essential component of risk stratification in these anomalies. Traditional methods include echocardiogram, single-photon emission computed tomography (SPECT), PET, CMR, or invasive FFR. If the patient does not have functional limitations, they can use an exercise bike or a treadmill. If not, agents like dobutamine or regadenoson can be used for pharmacologic stress. Physical stress testing is typically favored over pharmacologic because of the ability to simulate exercise or sports conditions, as reflected in the 2020 European Society of Cardiology Guidelines [12]. Based on the model of dynamic compression, vasodilatory drugs (regadenoson) may only evaluate the fixed component and not the dynamic component [13]. In negative testing, if the maximal heart rate is not achieved, this might provide false reassurance. This was evidenced by a case by Bigler et al. that showed a patient with a normal exercise SPECT at a heart rate of 156 beats per minute, followed by a positive dobutamine FFR at 172 beats per minute [14]. These authors suggest that the most accurate hemodynamic evaluation involves a dobutamine and volume challenge using IVUS and FFR to simulate 100% of the maximum heart rate [8]. Atropine can be given if the patient is still not achieving their maximal heart rate [8].

Treatment of these anomalies includes medical treatment, percutaneous intervention, or surgical revascularization. Medical treatment for AAOCA traditionally involves beta-blockers. In patients with AAOCA with an intramural course, coronary unroofing is carried out with the formation of a neo-ostium. Other procedures include coronary translocation or coronary artery bypass grafting in patients with CAD. While not carried out routinely, Angelini et al. demonstrated the potential effectiveness of stenting an anomalous RCA with symptomatic improvement [15]. Surgery is a Class 1 recommendation for AAOCA of the left or right coronary sinus with symptoms or evidence of ischemia attributed to the anomalous coronary artery [1].

While many coronary artery anomalies are discovered incidentally in asymptomatic patients, our anomalies were discovered while undergoing investigation for chest pain. In our first case, a congenital anomaly was not on the initial differential. Instead, it was identified incidentally on CT imaging. Given the high-risk features identified (interarterial/intramural course), exercise testing was used to determine the presence of ischemia. The patient could not reach his goal heart rate but was severely symptomatic and could not finish the test. In the setting of known high-risk features and symptomatic stress testing, conservative management was an unattractive option. After his unroofing procedure, his symptoms improved significantly. Our second patient presented with a non-ST elevation myocardial infarction. Angiography showed significant obstruction to flow during systole with little evidence of coronary artery disease. This was thought to be the culprit of his symptoms. CCTA was then completed to delineate other anatomical features and assist with surgical planning. The CCTA imaging was further helpful in identifying an interatrial and intramural course. Ultimately, the patient selected medical therapy and has been relatively asymptomatic at follow-up visits.

Autopsy studies suggest that R-AAOCA poses less risk of SCD than L-AAOCA [5,6]. However, these anomalies can still pose significant risks and be highly symptomatic in specific individuals, as demonstrated in our cases. While anomalous coronary arteries can be asymptomatic in specific individuals, it is crucial to consider these congenital variants in adults with angina or anginal equivalents. If a patient is suspected of having an anomalous coronary artery, obtaining a CCTA is an excellent initial imaging modality. In our cases, these scans were crucial in identifying high-risk anatomic features that gave further insight into the severity of these anomalies. If any of the aforementioned high-risk features are seen on imaging, clinicians should have a lower threshold to evaluate patients with non-invasive or invasive myocardial perfusion studies. If the myocardial perfusion testing is negative and the patient remains symptomatic, further testing may be considered until the maximum target heart rate can be achieved [8]. If this evaluation is positive, surgical intervention should be considered. In the future, percutaneous interventions may play a more significant role in therapeutic management.

## Conclusions

Coronary artery anomalies are some of the most frequently encountered congenital cardiovascular conditions. Anomalous coronary arteries may be discovered incidentally or during work-up in symptomatic patients. Some of these anomalies can be benign or malignant. However, R-AAOCA and L-AAOCA are difficult to risk stratify. R-AAOCA are encountered more frequently and typically cause fewer cases of SCD. However, as our cases demonstrate, they may cause significant morbidity. Due to their rarity, there is no clear consensus on the work-up and management of these conditions. We hope to share our experience with these cases to help future clinicians manage patients with anomalous right coronary arteries.
